# Evaluation of emergency obstetric and neonatal care services in Kumba Health District, Southwest region, Cameroon (2011–2014): a before-after study

**DOI:** 10.1186/s12884-020-2774-9

**Published:** 2020-02-11

**Authors:** Reine Suzanne Kadia, Benjamin Momo Kadia, Christian Akem Dimala, Desmond Aroke, Noel Vogue, Bruno Kenfack

**Affiliations:** 1 Global Health Systems Solutions, Douala, Littoral region Cameroon; 2 Faculty of Epidemiology and Population Health, London School of Hygiene and Tropical Medicine, London, UK; 3 Health and Human Development Research Network (2HD), Douala, Littoral region Cameroon; 4 Faculty of Medicine and Pharmaceutical Sciences, University of Dschang, Dschang, West region Cameroon

**Keywords:** Emergency, Obstetric, Neonatal, Care, Availability, Utilization, Quality

## Abstract

**Background:**

There is uncertainty regarding the status of emergency obstetric and neonatal care (EmONC) in the Cameroonian context where maternal and neonatal mortality are persistently high. This study sought to evaluate the coverage, functionality and quality of EmONC services in Kumba health district (KHD), the largest health district in Southwest Cameroon..

**Methods:**

A retrospective study of routine EmONC data for the periods 1 January 2011 to 31 December 2012 (when EmONC was being introduced) and 1 January 2013 to 31 December 2014 (when EmONC was fully instituted) was conducted. Coverage, functionality and quality of EmONC services were graded as per United Nations (UN) standards. Data was analysed using Epi-Info version 7 statistical software.

**Results:**

Among the 31 health facilities in KHD, 12 (39%) had been delivering EmONC services. Three (25%) of these were geographically inaccessible Among the 9 facilities that were assessed, 4 facilities (44%) performed designated signal functions, with 2 being comprehensive (CEmONC) and 2 basic (BEmONC). These exceeded the required minimum of 2.8 EmONC facilities/500000, 0.6 CEmONC facilities/500000 and 2.2 BEmONC facilities/500000, with reference to an estimated KHD population of 265,071. The signal functions that were least likely to be performed were neonatal resuscitation, manual evacuation of retained products and use of anticonvulsants. In 2011–2012, the facilities performed 35% of expected deliveries. This dropped to 28% in 2013–2014. Caesarean sections as a proportion of expected deliveries remained very low: 1.5% in 2010–2011 and 3.6% in 2013–2014. In 2011–2012, met needs were 6.8% and increased to 7.3% in 2013–2014. Direct obstetric fatality rates increased from 8 to 11% (*p* = 0.64). Intrapartum and very early neonatal deaths increased from 4.% to 7 (*p* = 0.89).

**Conclusion:**

Major gaps were observed in the performance of signal functions as well as the quality and utilization of EmONC. While the results of this study seem to indicate the need to sustainably scale up the utilization of quality EmONC, the interpretations of our findings require consideration of improvements in reporting of mortality data associated with the introduction of EmONC as well as dynamics in country-specific maternal health policies and the potential influence of these policies on EmONC indicators.

## Introduction

Worldwide, about 830 women die from complications of pregnancy and childbirth daily. Ninety-nine percent of these maternal deaths occur in low and middle-income countries and most are preventable [1]. In 2015, maternal mortality ratio was reported to be 239 per 100,000 life-births in low and middle-income countries [2], with the leading causes being direct obstetric complications notably, haemorrhage, hypertension, infections and unsafe abortion [3]. Sub-Saharan Africa alone accounts for 179,000 pregnancy and childbirth-related deaths each year [2]. In Cameroon, about 7000 women die from complications of pregnancy and childbirth yearly. The country had been ranked 15th from bottom on a global league table of maternal mortality ratios [4]. In 2000, 2005, 2010 and 2015, maternal mortality ratios in Cameroon were 750, 729, 676 and 596 respectively [5]. Thus, despite the enormous efforts made by the Cameroon Ministry of Health and its partners, the country had failed to attain its target of reducing maternal mortality ratio to 350 by the year 2015.

The main causes of maternal death (direct obstetric complications) can be prevented by a recommended package of nine evidence-based interventions (signal functions). These interventions which are collectively called emergency obstetric and newborn care (EmONC) services comprise: use of parenteral antibiotics, use of parenteral anticonvulsants, administration of parenteral uterotonics, manual removal of the placenta, removal of retained products of conception, assisted vaginal delivery, resuscitation of the newborn, emergency surgery (notably, emergency caesarean section) and blood transfusion. Comprehensive EmONC includes all signal functions while basic EmONC does not include emergency surgery and blood transfusion [6]. Basic EmONC only can help avert as much as 40% of intra-partum related newborn deaths and a considerable proportion of maternal deaths [7].

While the international scientific community acknowledges that quality EmONC services can significantly reduce maternal and neonatal deaths [8, 9], there has been much uncertainty regarding the status of these vital services in Cameroon. Maternal and neonatal mortality rates have been persistently high despite the introduction of EmONC, with national trends revealing considerable increase in mortality rates during the 2004 to 2011 period. Under these circumstances, it is imperative to assess and report the status of EmONC services in the country. This could reveal strategies of sustainably improving these essential services. This study aimed to assess coverage, functionality and quality of EmONC services in Kumba health district. This is one of the largest rural health districts in Cameroon and the largest in the Southwest region of the country. Because of the large size of the district and inadequate resources, it has been a challenge to the district health service to organize large-scale evaluations and generate comprehensive reports on the status of EMONC services. Consequently, since the introduction of EmONC services in the district, data on their availability, utilization and quality have remained obscure.

## Methods

### Study design, study period and setting

**Fig. 1:**
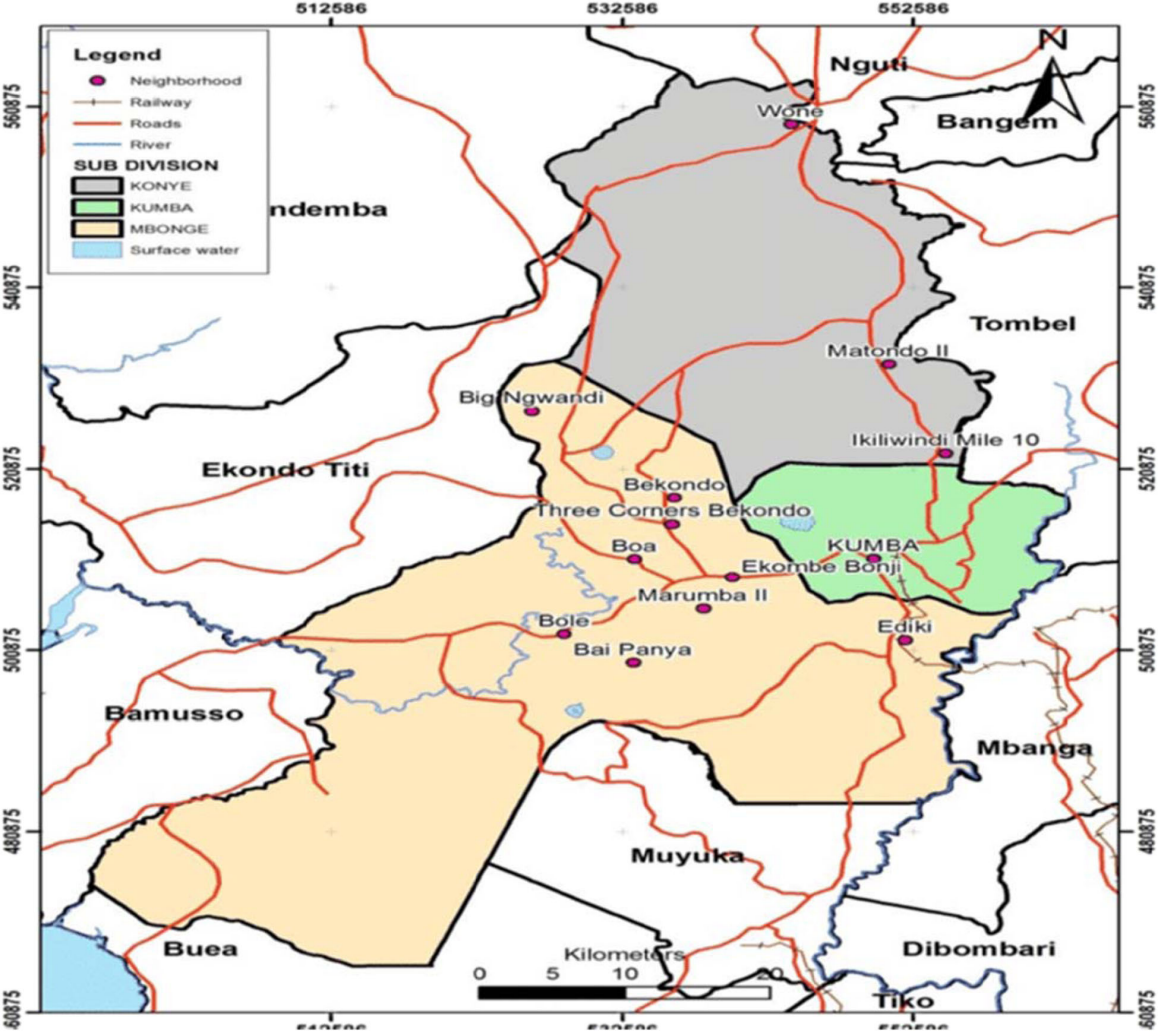
Map of Kumba health district, its health areas and boundaries

From June 1, 2015 to September 30, 2015 (4 months), a retrospective study of routine EmONC data recorded from 1st January 2011 to 31st December 2014 was conducted in health facilities in Kumba Health District (KHD). EmONC services were introduced in the district in 2011. KHD is the largest of the 18 health districts in the Southwest region of Cameroon. Figure 1 shows the Kumba health district. It spans from Kumba central sub-division, parts of Mbonge sub-division and parts of Ndian division. It is bounded to the north by Konye health district, to the west by Mbonge health district, to the south by Muyuka health district and to the east by the Littoral region whose capital is Douala. Kumba has a total surface area of 286 km^2^ and an estimated population of 265,071 inhabitants. This population was served by 31 health facilities distributed in 12 health areas.

### Selection of health facilities and data collection

Facilities with staff trained on delivering EmONC were eligible to participate in the study and they were identified through the District Health Service. Data were collected through facility-based survey. Locally-adapted data collection tools derived from the World Health Organization (WHO) handbook on emergency and obstetric care [6] were designed and pretested. The data collection tools comprised a questionnaire and a data collection guide. The questionnaire comprised a database review grid and an observational grid. The data base review grid was used to record the following information (obtained from the country’s most recent demographic health survey, the district health management information system and hospital health records where appropriate) for the period under review: the size and distribution of the population, a list of all health facilities (indicating those designated to provide EmONC), maternal and neonatal deaths, number of deliveries, obstetric complications and caesarean sections, performance of signal functions and utilization of EmONC services by pregnant women. The data obtained from this simple real time questionnaire (which was administered to unit heads in the facilities in parallel with direct observation of the performance of signal functions) enabled us to evaluate the performance of signal functions. The observational grid was used to record the following information through direct observation, interviews and registers in each facility: the type of facility (private or public), the category of the facility (integrated health centre, sub-divisional medical centre, or district hospital), and the availability of signal functions. Key indicators such as the frequency of post-partum haemorrhage, uterine rupture and prolonged labour were followed using the observational grid. These indicators allowed us to appreciate EmONC services and to appreciate the various causes of death.

### Measurement of outcomes

The outcomes in this study were the availability, utilization and quality of EmONC which were defined and graded as per United Nations (UN) standards (Table 1). Availability was graded using indicators 1 and 2, utilization was graded using indicators 3 to 5 and quality was reported using indicators 6 and 7.

**Table 1:** Emergency Obstetric and Neonatal Care Indicators

Indicators	Acceptable level
1. Availability of emergency obstetric care: basic and comprehensive care facilities	There are ≥5 EmONC facilities (including ≥1 comprehensive facility) for every 500,000 population.
2. Geographical distribution of emergency obstetric care facilities	Subnational areas have ≥5 EmONC facilities (including at least one comprehensive facility) for every 500,000 population.
3. Proportion of all births in emergency obstetric care facilities	Minimum acceptable level to be set locally
4. Met need for emergency obstetric care: proportion of women with major direct obstetric complications who are treated in such facilities	100% of women estimated to have major direct obstetric complications are treated in emergency obstetric care facilities.
5. Caesarean sections as a proportion of all births	Estimated proportion of births by caesarean section in the population is not < 5% or > 15%.
6. Direct obstetric case fatality rate	Case fatality rate among women with direct obstetric complications in EmONC facilities is < 1%.
7. Intra-partum and very early neonatal death rate	Standards to be determined locally
8. Proportion of maternal deaths due to indirect causes in emergency obstetric care facilities	No standard set.

Source: World Health Organization handbook on monitoring obstetric care [6]

### Statistical analysis

The data was divided for the time series January 1, 2011 to December 31, 2012 (when EmONC was introduced) and January 1, 2013 to December 31, 2014. Epi info version 7 statistical software was used for checking, coding, entering and analyzing the data. Frequency tables were used to present descriptive statistics. The t-test was used to compare means, proportions and rates before and after full EmONC. The level of statistical significance was set at *p* < 0.05.

### Ethical and administrative considerations

Ethical registry for this study was obtained from the Cameroon National Ethics Committee. Administrative authorization was obtained from the Southwest regional delegation for health, Kumba health district service and directors of participating health facilities.

## Results

**Fig. 2:**
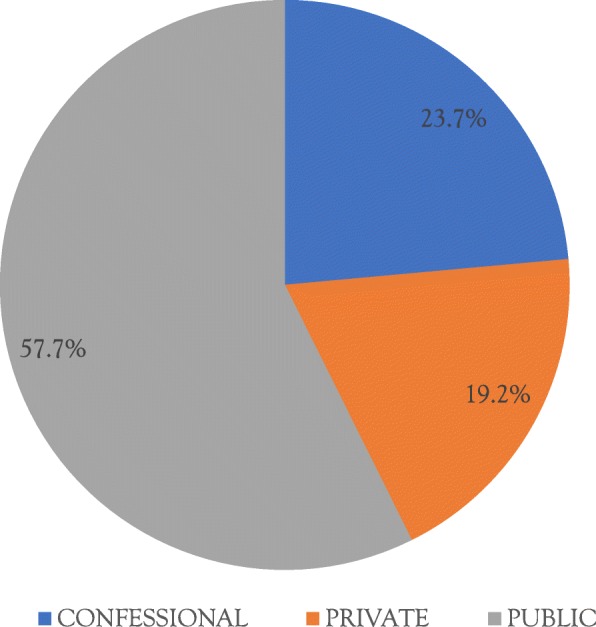
Types of health facilities practicing emergency obstetric and neonatal care in Kumba health district, 2011–2014

All health facilities in KDH were eligible for the study given that EmONC had been instituted in the district since 2011. However, only 12 (39%) of these had staff trained on EmONC. Among the 12 EmONC facilities, 3 (25%) were not evaluated because they were geographically inaccessible during the data collection phase: two facilities could not be accessed because of difficult road conditions during the rainy season while one facility could only be accessed using canoes which were hardly available. Among the 9 facilities that were evaluated, 58% were public facilities (Fig. 2). Among the assessed centres, 4(44%) had been performed designated signal functions, with 2 qualifying as CEmONC and 2 as BEmONC.

### Availability of facilities providing EmONC

**Fig. 3:**
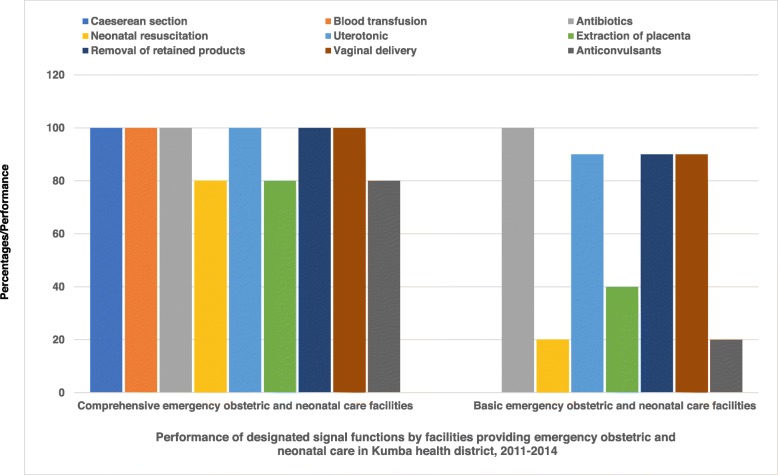
Performance of signal functions by facilities providing emergency obstetric and neonatal care services in Kumba health district, 2011–2014

The most lacking EmONC signal functions were assisted vaginal delivery, blood transfusion and cesarean section. Figure 3 shows the performance of signal functions for facilities qualifying as CEmONC and BEmONC. For the KHD population estimated at 265,071, the required UN minimum coverage was 2.8 EmONC facilities/500000, 2.2 BEmONC facilities/500000 and 0.6 CEmONC facilities/500000. For KHD, the 9 health facilities that were assessed met this minimum requirement since there were 4 EmONC facilities/265071, 2 CEmONC facilities/265071 and 2 BEmONC/265071.

### Utilization of EmONC services

**Fig. 4:**
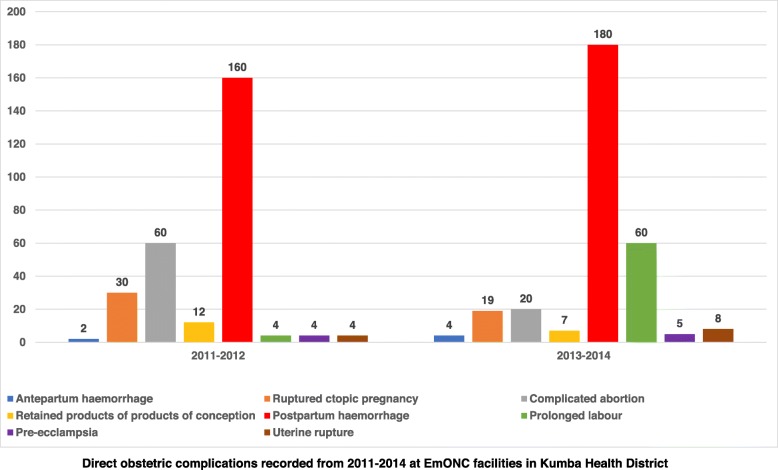
Direct obstetric complications recorded in facilities providing emergency obstetric and neonatal care services in Kumba health district, 2011–2014

In the 2011–2012 period, regarding the total deliveries expected for the two-year period, 7.% were carried out by BEmONC facilities and 27% by CEmONC facilities. In the 2013–2014 period, 11% of expected deliveries were performed by BEmONC facilities while CEmONC facilities performed just 10% of all expected deliveries. Thus, in 2011–2012, 35% of expected deliveries were done in EmONC facilities and in 2013–2014, 28% of expected deliveries were done in EmONC facilities. Caesarean section as a proportion of all births was 1.5% in 2011–2012 and increased to 4% in 2013–2014. In 2011–2012, met need was estimated at 7% and increased to 7% in 2013–2014.Over 90% of obstetric complications were not managed in EmONC facilities. Figure 4 compares direct obstetric complications recorded during the time segments.

### Quality of EmONC provided

Direct obstetric case fatality rate remained well above the recommended maximum of 1%. It increased from 8% in 2011–2012 to 11% in 2013–2014 (*p* = 0.64). Intrapartum and very early neonatal death rate increased from 2% and to 4% for CEmONC and decreased from 2.5 to 2.2% for BEmONC (i.e, overall increase from 4.% to 7, *p* = 0.89).

## Discussion

This study evaluated the availability, utilization and quality of EmONC in KHD, the largest health district in the Southwest region of Cameroon. The data assessed was for the period spanning from 2011 when EmONC was introduced in the district to the end of 2014, i.e., just before 2015 when the level of attainment of Millennium Development Goals had to be assessed. Although previous studies have evaluated EmONC services in sub-Saharan African settings, reports based on results from before-after studies or other quasi-experimental evaluation methods are scarce. According to this study, the number of facilities expected to provide EmONC services was slightly above the required UN minimum coverage. A considerable drop in deliveries in the included health facilities was noticed in the second segment of the period under review. Caesarean section rates remained very low. Met needs increased after EmONC were fully instituted. Direct obstetric case fatality rates remained high.

The availability of EmONC meeting the required minimum coverage in KHD is a pertinent deviation from the low availability which is characteristically reported in low-income settings especially during the period prior to the target Millennium Development Goal for maternal and newborn health [10–13]. Notwithstanding the apparently optimal number of facilities designated to provide EmONC in KHD, there seemed to be major gaps in the geographical distribution, performance of signal functions and quality of EmONC. Thus, the considerably high neonatal mortality rate observed in EmONC facilities was possibly linked with lack of the neonatal resuscitation signal function, but it is plausible that the full institution of EmONC also improved the recording of deaths. To be more certain about these links, there is need to conduct analytic studies comparing mortality rates between facilities without and facilities with EmONC (in general) and neonatal resuscitation (more specifically), and using appropriate statistical tests to assess if there is a statistically significant difference in mortality rates between the two types of facilities. However, this was not within the scope of this study which sought to investigate changes in EmONC indicators (including mortality data) before and after full implementation of EmONC. The discordance in availability of EmONC and quality of services observed in our study was also reported by Ameh et al in a large cross-sectional study evaluating the status of EmONC in six African countries [13]. A common lack of trained and qualified staff and lack of equipment to carry out signal functions could explain the discordance noted in our study and the report by Ameh et al. Large cross-sectional studies by Hanson et al in Tanzania [14], Wilunda et al in Uganda [15] and Abegunde et al in Nigeria [16] also revealed persistently high maternal and neonatal death rates even after the institution of EmONC. Contrary to these findings, *K*im et al in Afghanistan reported optimal EmONC quality with case fatality rates as low as 0.7% in their low-income study setting [17]. In line with these, there is evidence of a reduction in maternal and neonatal mortalities after ameliorations in training of personnel and performance of EmONC signal functions in a low-income context [18]. These contrary reports are possibly due to the higher level of technical and infrastructural facilities providing EmONC (Kim et al) and the scale-up of quality EmONC services (Ameh et al). In KHD, even though the lack of adequate resources may explain the increase in mortality after EmONC services were introduced, it is very likely that systems for registering and reporting mortality improved with the introduction of these vital services and this may partly explain the observed increased mortality after the introduction of EmONC. These suggest that scaling up quality EmONC in rural areas (like KHD) where most maternal and neonatal deaths occur ought to be be prioritized as a means of averting maternal and neonatal mortality and improving death reporting systems.

Some reports indicate serious short falls in EmONC indicators as the study by Admasu et al in Ethiopia [11]. These translated into negative outcomes such as low delivery rates, low caesarean section rates and high obstetric case fatality. Differences in population knowledge, attitudes and practices as well as differences in implementation gaps in different settings could explain these contrasting findings. Continuous sensitization of populations on the availability and effectiveness on EmONC and consistent monitoring of EmONC services by health authorities could alter behaviors and improve the status of EmONC services respectively in low-income settings. In our study, apart from the provision of EmONC, the increase in caesarean sections and overall deliveries as a proportion of expected deliveries could be also due to other factors such as obstetric kits whose pilot phase was launched by the ministry of health and the UNFPA by 2012 [19]. Obstetric kits lead to faster and cheaper access to obstetric care [19]. Moreover, healthcare providers inducing demand for obstetric services such as caesarean sections (as a result of reduced revenue associated with the policy of obstetric kits) is plausible especially in this low-income context.

This study is not void of limitations. There were reporting inconsistencies among the included facilities especially because of the retrospective nature of data collection. We tried to reduce the effect of this on the quality and internal validity of our results by introducing an observational grid which enabled direct observation of signal function performance although this mainly improved the quality of data for the second segment of the time series. The observational grid also helped to address this source of measurement bias by including data from the most standard sources that were available i.e., demographic health survey and district health information system. Furthermore, the records available did not allow us to consistently report other indicators of quality notably, indirect causes of maternal deaths. Nonetheless, we were able to capture data on direct obstetric case fatality rates and intrapartum and very early neonatal deaths which are very important indicators of EmONC. The findings of this study should be generalized with caution since three of the 12 health facilities that were eligible for inclusion in this study were not assessed.

## Conclusion

As theory of change, the burden of maternal and neonatal mortality is expected to significantly reduce if EmONC is available, of good quality and effectively utilized. For the period 2011–2014, KHD fully implemented EmONC during the period 2013–2014. This study found that albeit UN minimum coverage was met following the full institution of EmONC, there were major gaps in the performance of signal functions as well as the quality and utilization of EmONC. These possibly explain the persistently high maternal and neonatal mortality rates even though it can be argued that full implementation of EmONC also led to improvements in reporting of mortality data. The gaps in EmONC identified in this study suggest the urgent need to sustainably scale up quality and accessible EmONC services in KHD. This could re-enforce the quality of staff and equipment in EmONC facilities and contribute to sustainable improvements in EmONC. Notwithstanding the findings of this study, their interpretations should be made with consideration of dynamics in Cameroon’s maternal health policies and how these may influence indicators used in grading EmONC services. Further research is warranted to explore contextual factors that could influence the performance of signal functions and ultimately the functionality, quality and coverage of EmONC services in KHD.

## Data Availability

The data generated in this article will be made available on request by the corresponding author.

## Abbreviations

BEmONC: Basic Emergency Obstetric and Neonatal Care
CEmONC: Comprehensive Emergency Obstetric and Neonatal Care
EmONC: Emergency Obstetric and Neonatal Care
KHD: Kumba Health District
UN: United Nations
UNFPA: United Nations Population Fund
WHO: World Health Organization
